# Flowers, Ferns, and Ward Decorations

**Published:** 1886-10-02

**Authors:** 


### Flowers, Ferns, and Ward Decorations.

Communications for this column should be addressed, "Gardener," care of the Editor of The Hospital.

Everyone now admits the value and importance of cheerful surroundings for the sick. There are, however, many unnecessary difficulties which beset sisters and nurses as well as the higher officials in the hospitals, who earnestly labour to keep the wards and day-rooms bright with flowers and ferns throughout the year. The subjects of ward decoration, pictures in the wards, and other matters may be usefully discussed, and the Editor will be glad to receive suggestions and offers of assistance for this column. Inquiries are also invited, and every effort will be made to develop and assist all who are working to lighten the hours of suffering and to relieve the monotony of the patients who languish for weeks on the bed of suffering. A lady of great experience, who has a real love for flowers and ferns, and practical knowledge of their culture and management, will have charge of this department of The Hospital, and will do her best to aid any reader who desires information and assistance.

### The Flower Prize Essay.

A prize of three guineas will be awarded at Christmas to the writer of the best description of how to manage and maintain ferns and flowers in a hospital ward throughout the year. Full particulars of the plan of competition will be given in an early number.

